# Serologic Survey of IgG Against SARS-CoV-2 Among Hospital Visitors Without a History of SARS-CoV-2 Infection in Tokyo, 2020–2021

**DOI:** 10.2188/jea.JE20210324

**Published:** 2022-02-05

**Authors:** Takahiro Sanada, Tomoko Honda, Fumihiko Yasui, Kenzaburo Yamaji, Tsubasa Munakata, Naoki Yamamoto, Makoto Kurano, Yusuke Matsumoto, Risa Kohno, Sakiko Toyama, Yoshiro Kishi, Takuro Horibe, Yudai Kaneko, Mayumi Kakegawa, Kazushige Fukui, Takeshi Kawamura, Wang Daming, Chungen Qian, Fuzhen Xia, Fan He, Syudo Yamasaki, Atsushi Nishida, Takayuki Harada, Masahiko Higa, Yuko Tokunaga, Asako Takagi, Masanari Itokawa, Tatsuhiko Kodama, Michinori Kohara

**Affiliations:** 1 Department of Microbiology and Cell Biology, Tokyo Metropolitan Institute of Medical Science, Tokyo, Japan; 2 Department of Clinical Laboratory, and, Graduate School of Medicine the University of Tokyo Hospital, Tokyo, Japan; 3 Medical & Biological Laboratories Co., Ltd., Tokyo, Japan; 4 Laboratory for Systems Biology and Medicine, Research Center for Advanced Science and Technology, The University of Tokyo, Tokyo, Japan; 5 Suzhou Institute of Biomedical Engineering and Technology Chinese Academy of Sciences, Jiangsu, China; 6 The Key Laboratory for Biomedical Photonics of MOE at Wuhan National Laboratory for Optoelectronics – Hubei Bioinformatics & Molecular Imaging Key Laboratory, Systems Biology Theme, Department of Biomedical Engineering, College of Life Science and Technology, Huazhong University of Science and Technology, Hubei, China; 7 Reagent R&D Center, Shenzhen YHLO Biotech Co., Ltd., Guangdong, China; 8 Research Center for Social Science and Medicine, Tokyo Metropolitan Institute of Medical Science, Tokyo, Japan; 9 Center for Medical Research Cooperation, Tokyo Metropolitan Institute of Medical Science, Tokyo, Japan; 10 Schizophrenia Research Project, Tokyo Metropolitan Institute of Medical Science, Tokyo, Japan

**Keywords:** SARS-CoV-2, COVID-19, IgG seroprevalence, hospital visitors, Tokyo

## Abstract

**Background:**

Tokyo, the capital of Japan, is a densely populated city of >13 million people, so the population is at high risk of epidemic severe acute respiratory coronavirus 2 (SARS-CoV-2) infection. A serologic survey of anti–SARS-CoV-2 IgG would provide valuable data for assessing the city’s SARS-CoV-2 infection status. Therefore, this cross-sectional study estimated the anti–SARS-CoV-2 IgG seroprevalence in Tokyo.

**Methods:**

Leftover serum of 23,234 hospital visitors was tested for antibodies against SARS-CoV-2 using an iFlash 3000 chemiluminescence immunoassay analyzer (Shenzhen YHLO Biotech, Shenzhen, China) with an iFlash–SARS-CoV-2 IgG kit (YHLO) and iFlash–SARS-CoV-2 IgG-S1 kit (YHLO). Serum samples with a positive result (≥10 AU/mL) in either of these assays were considered seropositive for anti–SARS-CoV-2 IgG. Participants were randomly selected from patients visiting 14 Tokyo hospitals between September 1, 2020 and March 31, 2021. No participants were diagnosed with coronavirus disease 2019 (COVID-19), and none exhibited COVID-19-related symptoms at the time of blood collection.

**Results:**

The overall anti–SARS-CoV-2 IgG seroprevalence among all participants was 1.83% (95% confidence interval [CI], 1.66–2.01%). The seroprevalence in March 2021, the most recent month of this study, was 2.70% (95% CI, 2.16–3.34%). After adjusting for population age, sex, and region, the estimated seroprevalence in Tokyo was 3.40%, indicating that 470,778 individuals had a history of SARS-CoV-2 infection.

**Conclusions:**

The estimated number of individuals in Tokyo with a history of SARS-CoV-2 infection was 3.9-fold higher than the number of confirmed cases. Our study enhances understanding of the SARS-CoV-2 epidemic in Tokyo.

## INTRODUCTION

Coronavirus disease 2019 (COVID-19), a flu-like illness caused by severe acute respiratory syndrome coronavirus 2 (SARS-CoV-2), emerged in Central China in late 2019 and then rapidly spread worldwide. By the end of 2020, ≥80 million confirmed cases of SARS-CoV-2 infection had been reported worldwide, including ≥1.7 million deaths.^1^

Patients with COVID-19 develop a variety of clinical symptoms, which can include high fever, dry cough, fatigue, headache, myalgia, and diarrhea.^2^ In severe cases, pneumonia and dyspnea with hypoxemia are common and occasionally fatal. In contrast to severe cases, many SARS-CoV-2 infections are asymptomatic.^3^ Although these patients exhibit no clinical symptoms or signs, viral RNA can be detected in samples of saliva or nasopharyngeal and throat swabs. Both symptomatic and asymptomatic patients can potentially transmit the virus to others; thus, the ability to identify asymptomatic patients would aid in controlling COVID-19.

SARS-CoV-2 infection is confirmed based on detection of viral RNA using reverse transcription-polymerase chain reaction (RT-PCR) assays or detection of viral antigen using an antigen test.^4^ However, viral RNA or antigen is not always detectable in the period between infection and exclusion. Considering the possibility of asymptomatic patients and false-negative test results resulting from low levels of RNA or antigen in saliva or nasopharyngeal and throat swabs, the actual morbidity could be higher than the number of confirmed cases. To characterize the history of viral infections, serologic tests involving the detection of specific antibodies can be useful. Production of immunoglobulin G (IgG) that primarily recognizes the target nucleocapsid (N) or spike (S) protein of SARS-CoV-2 is induced approximately 7 days after symptom onset and maintained for at least 1 month.^5^ Generally, antibodies targeting the N protein are induced earlier than those targeting the S protein.^6^ In contrast, in the convalescent population, monitoring the anti-S IgG response was found to be a specific and sensitive means of identifying patients who had experienced SARS-CoV-2 infection.^7^ Analysis of such anti–SARS-CoV-2 antibodies would aid in determining the actual number of infections in a given time period.

The first case of COVID-19 in Japan was confirmed on January 16, 2020. The number of domestic infections increased gradually after that date, and by the end of March 2021, a total of 470,420 cases, including 9,159 deaths, had been reported in Japan.^8^ In Tokyo, Japan’s capital with a population of ≥13 million, the first case of COVID-19 was confirmed on January 24, 2020.^9^ By the end of March 2021, a total of 120,986 cases, including 1,815 deaths, had been confirmed in Tokyo, the most among all prefectures in Japan. As indicated above, these confirmed cases were diagnosed based on RT-PCR assays or antigen testing, but the data did not appear to reflect the actual number of SARS-CoV-2 infection cases.

The present study sought to clarify details of the SARS-CoV-2 epidemic in Tokyo by assessing the anti–SARS-CoV-2 antibody status of ≥23,000 serum samples collected from patients who visited 14 hospitals in Tokyo but exhibited no clinical signs associated with COVID-19.

## METHODS

### Ethics approval

This cross-sectional study was approved by the review board of the Tokyo Metropolitan Institute of Medical Science (approval no: 20-31).

### Study participants

**Figure 1.**
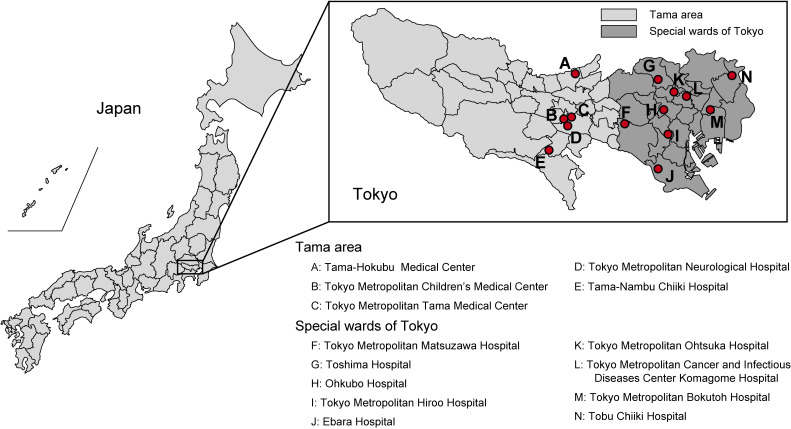
Location of the 14 hospitals in Tokyo, Japan. Five hospitals are located in the Tama (suburban) area, and nine hospitals are located in special wards of Tokyo (central area).

An opt-out recruitment approach (patients received written information about this study and were able to act on the information to decline participation) was used. Participants were randomly selected from outpatients visiting 14 Tokyo hospitals (eight metropolitan hospitals and six hospitals of the Tokyo Metropolitan Health and Hospitals Corporation) between September 1, 2020 and March 31, 2021 (Figure 1 and Table 1). Five hospitals are located in the Tama (suburban) area of the city, and nine hospitals are located in special wards of Tokyo (central area). Via medical interview, physicians confirmed that none of the participants were diagnosed with COVID-19, and none exhibited any COVID-19-related symptoms at the time of blood collection. Participants were guaranteed anonymity, and only participant demographic data, including age and gender, were collected.

**Table 1.** Characteristics and the number of study participants of each hospital

Hospital	Characteristic	Number of study participants
Special wards of Tokyo		15,324
Tokyo Metropolitan Matsuzawa Hospital	A center for psychiatric care in Tokyo	2,180
Toshima Hospital	Core hospital in northwest part of special wards	2,516
Ohkubo Hospital	Core hospital in western part of special wards	993
Tokyo Metropolitan Hiroo Hospital	An emergency and disaster medical center	1,260
Ebara Hospital	Core hospital in southern part of special wards	910
Tokyo Metropolitan Ohtsuka Hospital	A center for perinatal and pediatric care in Tokyo	1,620
Tokyo Metropolitan Cancer and Infectious Diseases Center Komagome Hospital	A center for cancer and infectious disease care in Tokyo	1,965
Tokyo Metropolitan Bokutoh Hospital	A comprehensive hospital in eastern Tokyo	2,200
Tobu Chiiki Hospital	A core hospital in the eastern part of Tokyo	1,680

Tama area		7,910
Tama-Hokubu Medical Center	Core hospital in the North Tama area	3,078
Tokyo Metropolitan Children’s Medical Center	A center for pediatric care in Tokyo	535
Tokyo Metropolitan Tama Medical Center	Core hospital in the Tama area	2,080
Tokyo Metropolitan Neurological Hospital	A center of the treatment of intractable neurological diseases	377
Tama-Nambu Chiiki Hospital	Core hospital in the South Tama area	1,840

### Measurement of anti–SARS-CoV-2 antibody levels

Serum samples left over after clinical testing were assayed to determine the concentration of anti–SARS-CoV-2 IgG using an iFlash 3000 chemiluminescence immunoassay analyzer (Shenzhen YHLO Biotech, Shenzhen, China) with an iFlash–SARS-CoV-2 IgG kit and iFlash–SARS-CoV-2 IgG-S1 kit. These kits were purchased from Shenzhen YHLO Biotech. The iFlash–SARS-CoV-2 IgG kit detects IgG specific for the both N and S proteins (YHLO IgG), but it primarily detects anti-N antibodies (eFigure 1).^10^ The iFlash–SARS-CoV-2 IgG-S1 kit detects IgG specific for the S1 subunit of the S protein (YHLO S1-IgG). According to the manufacturer’s instructions, ≥10 arbitrary units (AU)/mL is considered positive. The sensitivity of the iFlash–SARS-CoV-2 IgG kit is 63.6% for RT-PCR-positive patients 9 to 10 days after symptom onset and 100% for RT-PCR-positive patients ≥15 days after symptom onset, whereas the specificity of the kit is 100% for samples from RT-PCR-negative patients.^11^ Parai et al reported sensitivity and specificity values of 76.9% and 100%, respectively, for the iFlash–SARS-CoV-2 IgG kit.^12^ Kittel et al also reported sensitivity and specificity values of 77% and 100%, respectively, for the iFlash–SARS-CoV-2 IgG kit.^13^ The sensitivity of the iFlash–SARS-CoV-2 IgG-S1 kit was 19.7% for RT-PCR-positive patients 1 to 7 days after symptom onset, 67.6% for RT-PCR-positive patients 8 to 14 days after symptom onset and 98.3% for RT-PCR-positive patients ≥15 days after symptom onset, and the specificity of the iFlash–SARS-CoV-2 IgG-S1 kit was 100% for RT-PCR-negative samples (eFigure 2). As both assays have a specificity of 100%, serum samples with a positive result (≥10 AU/mL) in assays using the iFlash–SARS-CoV-2 IgG and/or iFlash–SARS-CoV-2 IgG-S1 kit were considered seropositive for anti–SARS-CoV-2 IgG.

### Estimating the number of individuals with a history of SARS-CoV-2 infection in Tokyo

The number of individuals in Tokyo with a history of SARS-CoV-2 infection was estimated from the age-, gender-, and region-specific seroprevalence of anti–SARS-CoV-2 IgG, according to 2020 statistics of resident registration^14^ (eTable 1). Estimation of the number of individuals in special wards and areas other than special wards with a history of SARS-CoV-2 infection was based on the seroprevalence of anti–SARS-CoV-2 IgG in special wards and Tama area, respectively.

### Statistical analyses

An exact binomial distribution was used to calculate 95% confidence intervals (CIs). Associations between variables and the presence of anti-SARS-CoV-2 IgG were evaluated using the chi-squared test, and adjusted residuals were then calculated. Multivariable analysis was conducted with logistic regression to adjust for sex and region to evaluate associations between age and seroprevalence of anti–SARS-CoV-2 IgG. Statistical analyses were performed using R software, version 4.0.3 (R Foundation for Statistical Computing, Vienna, Austria), and *P* values <0.05 were considered significant.

## RESULTS

### General characteristics of study participants

Table 2 summarizes the general demographic characteristics of the study participants. None of the participants had a previous diagnosis of COVID-19, and no participants exhibited any COVID-19-related symptoms at the time of blood collection. Blood samples from approximately 3,000 participants per month, and a total of 23,234 participants were collected. The 23,234 study participants included 11,553 (49.7%) males and 11,681 (50.3%) females. The age range was 0 to 103 years. The median age was 69 (interquartile range, 53–78) years. A total of 15,324 and 7,910 participants were from hospitals located in special wards and the Tama area, respectively.

**Table 2.** General characteristics of study participants

		2020				2021			Total
	
		September	October	November	December	January	February	March	
Participants		3,837	4,006	3,438	3,152	3,056	2,711	3,034	23,234

Sex	Male	1,933	1,986	1,732	1,534	1,512	1,359	1,497	11,553
	Female	1,904	2,020	1,706	1,618	1,544	1,352	1,537	11,681

Age, years	≤19	99	114	118	103	95	81	71	681
	20–39	317	408	311	320	281	213	295	2,145
	40–59	829	928	753	715	694	586	665	5,170
	60–79	1,770	1,771	1,556	1,413	1,352	1,226	1,389	10,477
	≥80	822	785	700	601	634	605	614	4,761

Region	Special wards of Tokyo	2,415	2,653	2,337	2,157	1,995	1,741	2,026	15,324
	Tama area	1,422	1,353	1,101	995	1,061	970	1,008	7,910

### Seroprevalence of anti–SARS-CoV-2 IgG

**Figure 2.**
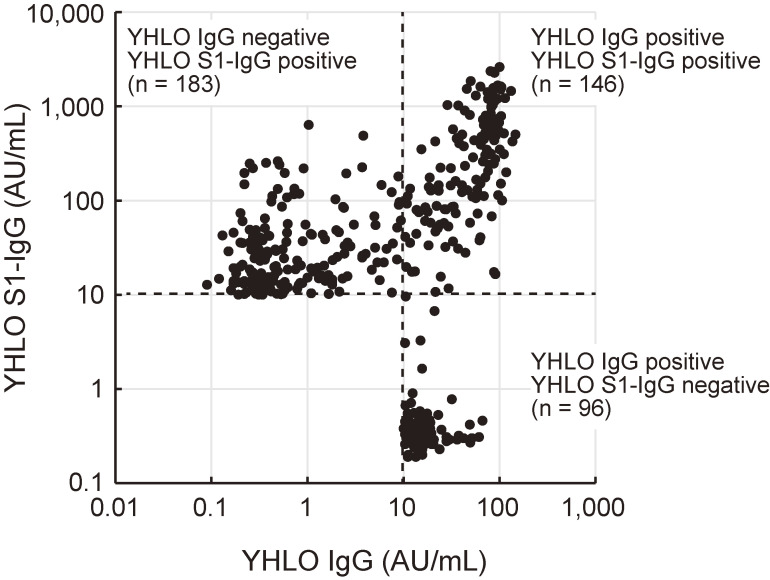
Scatterplot of the levels of YHLO IgG and S1-IgG. Each dot represents the level of YHLO IgG (x-axis) and YHLO S1-IgG (y-axis) for an individual positive for YHLO IgG and/or S1-IgG. Broken lines indicate the cutoff value for the IgG level (10 arbitrary units [AU]/mL).

Of 23,234 study participants, 425 participants were positive for anti–SARS-CoV-2 IgG (YHLO IgG and/or S1-IgG) (Figure 2 and eTable 2), and the overall seroprevalence of anti–SARS-CoV-2 IgG for the entire study population was 1.83% (95% CI, 1.66–2.01%) (Table 3). Of 425 participants positive for anti–SARS-CoV-2 IgG, a total of 242, 329, and 146 participants were positive for YHLO IgG, YHLO S1-IgG, and both YHLO IgG and S1-IgG, respectively.

**Table 3.** Univariable and multivariable analysis of factors associated with positive result for IgG antibodies

		Number of participants	Seroprevalence (95% CI)	Univariable anlysis		Multivariable analysis	
	
				OR (95% CI)	*P* value	OR (95% CI)	*P* value
Overall		23,234	1.83% (1.66–2.01)				
Sex					0.073		0.11
	Male	11,553	1.67% (1.44–1.92)	0.84 (0.69–1.02)		0.85 (0.70–1.03)	
	Female	11,681	1.99% (1.74–2.26)	Ref		Ref	
Age, years					0.00053		0.0012
	≤19	681	1.03% (0.41–2.11)	0.86 (0.39–1.89)		0.96 (0.43–2.12)	
	20–39	2,145	2.42% (1.82–3.17)	2.05 (1.40–3.00)		1.94 (1.32–2.84)	
	40–59	5,170	2.11% (1.73–2.54)	1.78 (1.29–2.45)		1.74 (1.26–2.40)	
	60–79	10,477	1.91% (1.66–2.19)	1.61 (1.19–2.16)		1.61 (1.20–2.16)	
	≥80	4,761	1.20% (0.91–1.55)	Ref		Ref	
Region					0.014		0.048
	Special wards	15,324	1.98% (1.77–2.22)	1.30 (1.05–1.61)		1.24 (1.00–1.54)	
	Tama area	7,910	1.53% (1.27–1.83)	Ref		Ref	

CI, confidence interval; OR, odds ratio; Ref, reference.

The seroprevalence of anti–SARS-CoV-2 IgG among males and females who participated in this study was 1.67% (95% CI, 1.44–1.92%) and 1.99% (95% CI, 1.74–2.26%), respectively, and there was no significant difference between the sex subgroups (Table 3). The seroprevalence of anti–SARS-CoV-2 IgG among participants in the ≤19, 20–39, 40–59, 60–79, and ≥80 year age groups for the entire study group was 1.03% (95% CI, 0.41–2.11%), 2.42% (95% CI, 1.82–3.17%), 2.11% (95% CI, 1.73–2.54%), 1.91% (95% CI, 1.66–2.19%), and 1.20% (95% CI, 0.91–1.55%), respectively. Among participants categorized by the region of hospital location, the seroprevalence of anti–SARS-CoV-2 IgG was 1.98% (95% CI, 1.77–2.22%) in special wards (central area) and 1.53% (95% CI, 1.27–1.83%) in the Tama area (suburban area). Univariable analysis and multivariable logistic regression analyses adjusting for sex and hospital region indicated that the seroprevalence of anti–SARS-CoV-2 IgG was significantly higher at hospitals in the special wards than the Tama area, and the seroprevalence of anti–SARS-CoV-2 IgG was significantly lower among patients ≥80 years of age.

Table 4 summarizes the seroprevalence of YHLO IgG, YHLO S1-IgG, and both YHLO IgG and S1-IgG. The overall seroprevalence of YHLO IgG, YHLO S1-IgG, and both YHLO IgG and S1-IgG among all study participants was 1.04% (95% CI, 0.92–1.18%), 1.42% (95% CI, 1.27–1.58%), and 0.63% (95% CI, 0.53–0.74%), respectively. Females exhibited significantly higher seroprevalence of YHLO IgG than males. The seroprevalence of YHLO S1-IgG and both YHLO IgG and S1-IgG was significantly increased among patients 20–39 years of age. The seroprevalence of YHLO IgG, YHLO S1-IgG, and both YHLO IgG and S1-IgG was significantly decreased among patients ≥80 years of age. In terms of hospital location, the seroprevalence of YHLO IgG, YHLO S1-IgG, and both YHLO IgG and S1-IgG was significantly higher at hospitals in the special wards than the Tama area.

**Table 4.** Seroprevalence of IgG antibodies against SARS-CoV-2

		Number of participants	YHLO IgG	YHLO S1-IgG	YHLO IgG and YHLO S1-IgG
		
			Seroprevalence (95% CI)	Seroprevalence (95% CI)	Seroprevalence (95% CIl)
Overall		23,234	1.04% (0.92–1.18)	1.42% (1.27–1.58)	0.63% (0.53–0.74)
Sex			*P* = 0.018	*P* = 0.24	*P* = 0.078
	Male	11,553	0.88% (0.72–1.07)	1.32% (1.12–1.55)	0.54% (0.41–0.69)
	Female	11,681	1.20% (1.01–1.41)	1.51% (1.29–1.74)	0.72% (0.57–0.89)
Age, years			*P* < 0.001	*P* < 0.001	*P* < 0.001
	≤19	681	0.88% (0.32–1.91)	0.44% (0.09–1.28)	0.29% (0.04–1.06)
	20–39	2,145	1.31% (0.87–1.88)	2.33% (1.73–3.06)	1.21% (0.79–1.77)
	40–59	5,170	1.49% (1.18–1.86)	1.64% (1.32–2.03)	1.03% (0.77–1.34)
	60–79	10,477	0.96% (0.79–1.17)	1.48% (1.26–1.73)	0.53% (0.40–0.69)
	≥80	4,761	0.63% (0.43–0.90)	0.76% (0.53–1.05)	0.19% (0.09–0.36)
Region			*P* = 0.025	*P* = 0.007	*P* = 0.006
	Special wards	15,324	1.15% (0.99–1.33)	1.57% (1.38–1.78)	0.73% (0.60–0.88)
	Tama area	7,910	0.83% (0.65–1.06)	1.13% (0.90–1.38)	0.43% (0.30–0.60)

CI, confidence interval.

### Estimated anti–SARS-CoV-2 IgG seroprevalence in Tokyo

**Figure 3.**
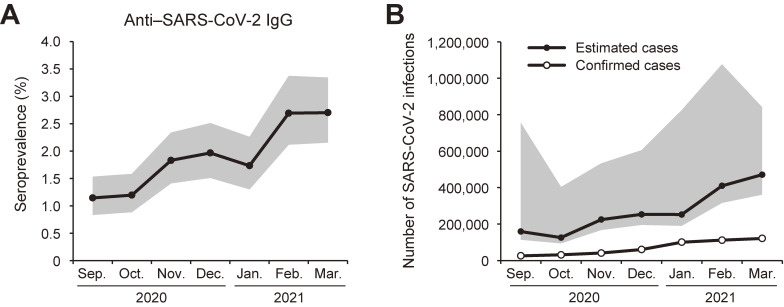
Seroprevalence of anti–SARS-CoV-2 IgG. (A) Prevalence of anti–SARS-CoV-2 IgG. Gray area indicates 95% confidence interval. (B) Number of individuals infected with SARS-CoV-2 as estimated from the adjusted seroprevalence, and cumulative number of confirmed SARS-CoV-2 cases in Tokyo by the end of each month. Gray area indicates 95% confidence interval of estimated cases.

The seroprevalence of anti–SARS-CoV-2 IgG was 1.15% (95% CI, 0.83–1.54%) in September 2020 and then gradually increased (Figure 3A). The seroprevalence in October, November, and December 2020, and January, February, and March 2021 was 1.20% (95% CI, 0.88–1.59%), 1.83% (95% CI, 1.41–2.34%), 1.97% (95% CI, 1.51–2.51%), 1.73% (95% CI, 1.30–2.26%), 2.69% (95% CI, 2.12–3.37%), and 2.70% (95% CI, 2.16–3.34%), respectively.

Based on the seroprevalence of anti–SARS-CoV-2 IgG determined in this study, we estimated the number of Tokyo residents positive for anti–SARS-CoV-2 IgG by adjusting the seroprevalence data based on the age and sex structure of the local population (eTable 3). The adjusted seroprevalence of anti–SARS-CoV-2 IgG in September, October, November, and December 2020, and January, February, and March 2021 was 1.15% (95% CI, 0.82–5.46%), 0.91% (95% CI, 0.68–2.91%), 1.62% (95% CI, 1.21–3.86%), 1.83% (95% CI, 1.41–4.38%), 1.82% (95% CI, 1.38–5.96%), 2.96% (95% CI, 2.28–7.78%), and 3.40% (95% CI, 2.61–6.08%), respectively. The estimated number of Tokyo residents positive for anti–SARS-CoV-2 IgG in September, October, November, and December 2020, and January, February, and March 2021 was 158,743 (95% CI, 113,580–755,617), 125,539 (95% CI, 93,938–403,003), 224,769 (95% CI, 167,660–533,411), 253,011 (95% CI, 194,954–605,747), 252,139 (95% CI, 190,345–824,608), 409,659 (95% CI, 316,043–1,076,318), and 470,778 (95% CI, 361,100–841,226), respectively (Figure 3B). By the end of March 2021, there were 120,986 cumulative confirmed cases as determined using RT-PCR or antigen testing. These data suggest that there were 3.9-fold (95% CI, 3.0–7.0–fold) more cases of SARS-CoV-2 infection than confirmed cases.

## DISCUSSION

A number of nations are currently facing difficult public health issues surrounding the COVID-19 pandemic. Elucidating details regarding the prevalence and spread of SARS-CoV-2 is thus essential to controlling COVID-19. In this study, we determined the levels of anti–SARS-CoV-2 IgG in randomly selected participants who visited hospitals in Tokyo, Japan. Blood samples from approximately 3,000 participants per month and a total of 23,234 participants were analyzed. Our data indicate that the estimated seroprevalence of anti–SARS-CoV-2 IgG was 3.40% in Tokyo in March 2021. By the end of March 2021, a total of 120,986 patients in Tokyo had been diagnosed with SARS-CoV-2 infection using RT-PCR or antigen testing in Tokyo; thus, the estimated number of Tokyo residents infected with SARS-CoV-2 was 3.9-fold higher than the number of confirmed cases, which suggests that 74.3% of infections were undiagnosed. In Japan, a cluster-based approach strategy was conducted.^15^ This strategy was based on a retrospective investigation to identify source cases and super-spreading clusters. Thus, diagnosis of SARS-CoV-2 infection is primarily reserved for patients suspected as having COVID-19 based on clinical signs or for individuals who were strongly suspected of having COVID-19 because of close contact with infected patients. This approach might be successful in the beginning of a pandemic. However, as the number of infected patients increases, it becomes too difficult to conduct retrospective contact tracing for all cases. Additionally, in this approach, most asymptomatic cases have not been tested for diagnosis of COVID-19. Thus, the actual number of cases of infection is probably underestimated.

In this study, we used two antibody detection kits with high specificity and combined the results. Although the iFlash–SARS-CoV-2 IgG kit detects anti-N and anti-S antibodies (YHLO IgG), it primarily detects anti-N antibodies (eFigure 1).^10^ According to the manufacturer, this could be because the magnetic beads are coated predominantly with the N protein antigen, with only a small proportion of S protein. To detect anti–SARS-CoV-2 antibodies with high sensitively and accuracy, serum samples were also tested for anti-S1 IgG (YHLO S1-IgG). As the sensitivity of these two kits is not 100%, but their specificity is 100%, we used two kits and combined the results to detect anti–SARS-CoV-2 IgG more sensitively. Indeed, of 425 participants positive for YHLO IgG and/or S1-IgG, 279 were seropositive for only YHLO IgG or YHLO S1-IgG. Although the sensitivity increased using both kits, the actual sensitivity was not determined, and we must take into account underestimation of the seroprevalence. Though further analysis is required, it appears that using two kits would be an effective means of accurately detecting anti–SARS-CoV-2 IgG.

A number of seroprevalence surveys have been reported for countries where intense SARS-CoV-2 outbreaks occurred in 2020. For example, the reported seroprevalence in Italy (March–April 2020), Spain (April–May 2020), England (June–July 2020), and the USA (July 2020) was 11.0%, 5.0%, 6.0%, and 8.0%, respectively.^16^^–^^19^ Tokyo, the capital of Japan, is one of the largest cities in the world and has the highest population density in Japan. As such, Tokyo is at increased risk of epidemics. Although the number of diagnosed cases was announced daily by the Tokyo Metropolitan Government, there are few reports regarding the seroprevalence of anti–SARS-CoV-2 IgG in Tokyo or the Tokyo metropolitan area. Our results indicate that the seroprevalence of anti–SARS-CoV-2 IgG increased gradually from September 2020 to March 2021. The seroprevalence in January 2021 declined from the previous month’s result, but not significantly. In March 2021, the seroprevalence reached 2.70%. Previously, Takita et al reported an overall seroprevalence of anti–SARS-CoV-2 IgG of 3.83% in Tokyo between April and May 2020,^20^ higher than our results. This discrepancy could have been due to differences in serological tests used. Takita et al used a rapid immunochromatographic antibody test. The reported specificity of the antibody test used in their study was 95.7% for iFlash–SARS-CoV-2 IgG kit-negative samples.^21^ A high incidence of false-positive rapid immunochromatographic test results in patients with common human coronavirus pneumonia has also been reported.^22^ For these reasons, the anti–SARS-CoV-2 IgG seroprevalence of our study would be lower than that of the previous study. Consistent with our results, Takita et al also reported that the seroprevalence was significantly higher in central Tokyo (special wards) than suburban areas of Tokyo. These results suggest that SARS-CoV-2 infections occurred primarily in central Tokyo. Our study is the first seroprevalence survey conducted in Japan involving more than 20,000 participants. Our results will help elucidate details of the SARS-CoV-2 epidemic in Tokyo and other areas of Japan.

A limitation of this study is participant bias. The study participants were enrolled from patients visiting hospitals. Thus, elderly patients constituted the majority of the study population. In addition, behavior patterns would be expected to differ between individuals who visit the hospital and those who do not. Indeed, the ≤19 years of age group included the fewest participants among all age groups examined, which could have affected the low IgG seroprevalence in the ≤19 years of age group and the wide 95% CI of estimated cases. Additionally, as the participating hospitals are large, patients were likely to come from various areas. Thus, participants in this study might not reflect the local population. For a more accurate analysis, a study involving cooperation of community-based clinics and a wider variety of participants, including healthy volunteers, would be required.

As described above, differences in serologic tests, participant characteristics, and study population size can affect seroprevalence estimates; thus, it is difficult to directly compare our results with those of other studies. Continuous surveys conducted in the same manner would provide more information to help elucidate dynamic changes in the number of SARS-CoV-2 infections and antibody kinetics in subclinical infections. In addition, constant monitoring of the subclinical infection rate would enhance understanding of changes in virus pathogenicity. Additionally, as vaccination in Japan began in 2021, such surveys will be useful tools for evaluating herd immunity. Herd immunity is a key concept in strategies to control COVID-19. The Delta (B.1.617.2) variants showed high transmissibility and replaced all other SARS-CoV-2 variants in Japan. Based on the estimated basic reproduction number (R_0_) for Delta of 5.08, according to the study of Liu et al,^23^ herd immunity is achieved when over 80% of a population acquires immunity to SARS-CoV-2.^24^ Because of decline in antibody titer or poor immune response after vaccination and non-seroconversion after SARS-CoV-2 infection,^25^^,^^26^ it is difficult to predict the actual proportion of the population with immunity to SARS-CoV-2 from the number of vaccinated and infected people. To control COVID-19, it is important to understand the immune status of the population.

In conclusion, we assessed the seroprevalence of anti–SARS-CoV-2 IgG among 23,234 visitors to Tokyo-area hospitals from September 2020 through March 2021. The overall seroprevalence of anti–SARS-CoV-2 IgG was 1.83% (95% CI, 1.66–2.01%) for the entire study population, and the seroprevalence in March 2021 was 2.70% (95% CI, 2.16–3.34%). After adjusting for age and sex based on the local population, the estimated seroprevalence in Tokyo was 3.40% (95% CI, 2.61–6.08%), indicating that 470,778 (95% CI, 361,100–841,226) Tokyo residents had a history of SARS-CoV-2 infection. Our study enhances understanding of the epidemic in Tokyo and the means of controlling the spread of SARS-CoV-2.

## APPENDIX A. SUPPLEMENTARY DATA

eTable 1. Population distribution of Tokyo, according to 2020 statistics of resident registration

eTable 2. The number of study participant and participants positive for anti–SARS-CoV-2 IgG

eTable 3. Estimated cases of each subgroup based on anti–SARS-CoV-2 IgG seroprevalence

eFigure 1. Correlation of YHLO IgG level with anti-N IgG (a) or YHLO S1-IgG (b)

Correlation of YHLO IgG level with anti-N IgG (a) or YHLO S1-IgG (b). Each dot represents the level of YHLO IgG (x-axis), anti-N IgG (a, y-axis), or YHLO S1-IgG (b, y-axis) for an individual positive for YHLO IgG and/or YHLO S1-IgG. Red lines indicate regression curves. AU, Arbitrary units.

eFigure 2. YHLO S1-IgG levels in th e PCR-negative and PCR-positve subjects

Each dot represents the level of YHLO S1-IgG in PCR-negative subjects (n = 163), PCR-positive subjects 1 to 7 days after symptom onset (n = 122), PCR-positive subjects 8 to 14 days after symptom onset (n = 340), and PCR-positive subjects ≥15 days after symptom onset (n = 423).

Broken line indicates the cutoff value (10 arbitrary units [AU]/mL).
