# Supplementary material for: Serologic Survey of IgG Against SARS-CoV-2 Among Hospital Visitors Without a History of SARS-CoV-2 Infection in Tokyo, 2020–2021
Source: J Epidemiol. 2022 Feb 5;32(2):105–11. doi: 10.2188/jea.JE20210324 (PMC8761565; doi:10.2188/jea.JE20210324)
Supplement: Supplementary file 1 [file je-32-105-s001.pdf]

eTable 1. Population distribution of Tokyo, according to 2020 statistics of resident registration

		Male	Female	Total
Special wards of Tokyo				
Age, years	≤19	732,622	701,307	1,433,929
	20–39	1,378,421	1,360,129	2,738,550
	40–59	1,469,327	1,420,432	2,889,759
	60–79	891,110	965,006	1,856,116
	≥80	227,921	424,334	652,255
Total		4,699,401	4,871,208	9,570,609
Areas other than special wards				
Age, years	≤19	369,408	350,251	719,659
	20–39	502,322	477,140	979,462
	40–59	649,090	614,707	1,263,797
	60–79	459,203	508,733	967,936
	≥80	125,877	207,584	333,461
Total		2,105,900	2,158,415	4,264,315
Tokyo				
Age, years	≤19	1,102,030	1,051,558	2,153,588
	20–39	1,880,743	1,837,269	3,718,012
	40–59	2,118,417	2,035,139	4,153,556
	60–79	1,350,313	1,473,739	2,824,052
	≥80	353,798	631,918	985,716
Total		6,805,301	7,029,623	13,834,924

eTable 2. The number of study participant and participants positive for anti–SARS-CoV-2 IgG

		September 2020		October 2020		November 2020		December 2020		January 2021		February 2021		March 2021	
		Positive	Tested	Positive	Tested	Positive	Tested	Positive	Tested	Positive	Tested	Positive	Tested	Positive	Tested
**Special wards of Tokyo**															
Male															
Age, years	≤19	0	7	0	13	0	16	0	11	0	4	0	5	1	11
	20–39	2	88	0	115	3	86	2	86	2	70	2	45	3	85
	40–59	5	276	4	312	3	291	5	258	6	258	9	192	7	225
	60–79	7	616	10	622	14	576	6	514	5	478	9	437	10	479
	≥80	1	234	0	252	2	234	1	185	1	175	1	198	5	198
Female															
Age, years	≤19	0	3	0	13	0	10	0	9	0	6	0	3	0	11
	20–39	3	145	4	219	5	158	6	171	5	152	6	116	5	147
	40–59	5	292	6	336	3	268	9	269	7	241	8	205	12	236
	60–79	7	525	10	517	11	467	11	445	8	387	9	347	15	437
	≥80	0	229	3	254	3	231	6	209	4	224	3	193	4	197
**Tama area**															
Male															
Age, years	≤19	0	51	0	50	0	56	0	47	2	50	0	45	9	25
	20–39	0	25	0	25	0	21	0	24	0	24	0	22	0	18
	40–59	0	105	1	116	0	71	2	70	2	85	3	70	2	71
	60–79	2	331	5	338	8	255	5	234	4	242	11	233	1	264
	≥80	2	200	3	143	4	126	2	105	1	126	1	112	4	121
Female															
Age, years	≤19	0	38	0	38	2	36	0	36	0	35	2	28	0	24
	20–39	0	59	0	49	0	46	0	39	0	35	0	30	2	45
	40–59	2	156	0	164	1	123	1	118	1	110	2	119	4	133
	60–79	5	298	2	294	4	258	5	220	4	245	6	209	3	209
	≥80	3	159	0	136	0	109	1	102	1	109	1	102	2	98

eTable 3. Estimated cases of each subgroup based on anti–SARS-CoV-2 IgG seroprevalence

		Estimated cases (95% confidence interval)						
		September 2020	October 2020	November 2020	December 2020	January 2021	February 2021	March 2021
**Special wards of Tokyo**								
Male								
Age, years	≤19	0 (0–300,094)	0 (0–180,996)	0 (0–150,852)	0 (0–208,734)	0 (0–441,306)	0 (0–382,300)	66,602 (1,684–302,412)
	20–39	31,328 (3,810–109,856)	0 (0–43,514)	48,084 (9,997–135,879)	32,056 (3,899–112,334)	39,383 (4,796–137,055)	61,263 (7,483–208,821)	48,650 (10,116–137,424)
	40–59	26,618 (8,680–61,371)	18,838 (5,148–47,751)	15,148 (3,131–43,831)	28,475 (9,289–65,597)	34,170 (12,609–73,361)	68,875 (31,819–128,002)	45,712 (18,511–92,637)
	60–79	10,126 (4,082–20,738)	14,327 (6,893–26,169)	21,659 (11,897–36,041)	10,402 (3,828–22,486)	9,321 (3,034–21,601)	18,352 (8,429–34,517)	18,604 (8,961–33,913)
	≥80	974 (25–5,374)	0 (0–3,312)	1,948 (236–6,959)	1,232 (31–6,780)	1,302 (33–7,162)	1,151 (29–6,340)	5,756 (1,880–13,207)
Total		69,046 (35,634–381,397)	33,164 (17,587–221,951)	86,839 (45,690–264,375)	72,166 (37,444–299,254)	84,177 (42,918–538,062)	149.641 (83,572–564,021)	185,324 (104,409–442,187)
Female								
Age, years	≤19	0 (0–496,244)	0 (0–173,260)	0 (0–216,351)	0 (0–235,826)	0 (0–322,081)	0 (0–496,244)	0 (0–199,812)
	20–39	28,141 (5,831–80,617)	24,843 (6,798–62,705)	43,042 (14,082–98,341)	47,724 (17,659–101,743)	44,741 (14,642–102,137)	70,352 (26,136–148,505)	46,263 (15,145–105,532)
	40–59	24,322 (7,930–56,115)	25,365 (9,348–54,630)	15,900 (3,288–45,970)	47,524 (21,890–88,862)	41,257 (16,699–83,702)	55,431 (24,142–107,162)	72,225 (37,707–123,770)
	60–79	12,867 (5,189–26,323)	18,665 (8,988–34,048)	22,730 (11,402–40,293)	23,854 (11,969–42,265)	19,948 (8,652–38,913)	25,029 (11,510–46,960)	33,124 (18,660–54,023)
	≥80	0 (0–6,781)	5,012 (1,036–14,481)	5,511 (1,140–15,905)	12,182 (4,501–26,069)	7,577 (2,073–19,132)	6,596 (1,365–18,990)	8,616 (2,359–21,713)
Total		65,330 (36,600–565,579)	73,885 (47,586–254,537)	87,184 (53,343–313,428)	131,283 (89,316–377,805)	113,524 (72,696–444,168)	157,408 (101,335–663,052)	160,228 (111,154–376,336)
All		134,376 (90,310–724,132)	107,049 (76,483–368,345)	174,023 (120,745–461,608)	203,449 (148,981–538,624)	197,702 (139,656–759,250)	307,049 (220,392–960,797)	345,552 (250,919–681,232)
**Areas other than special wards**								
Male								
Age, years	≤19	0 (0–25,776)	0 (0–26,273)	0 (0–23,550)	0 (0–27,885)	14,776 (1,803–50,660)	0 (0-29,074)	0 (0-50,677)
	20–39	0 (0–68,911)	0 (0–68,911)	0 (0–80,923)	0 (0–71,568)	0 (0–71,568)	0 (0-77,545)	55,814 (6,908-174,366)
	40–59	0 (0–22,408)	5,596 (142–30,569)	0 (0–32,863)	18,545 (2,258–64,538)	15,273 (1,858–53,500)	27,818 (5,794-78,007)	9,142 (231-49,327)
	60–79	2,775 (336–9,944)	6,793 (2,213–15,697)	14,406 (6,263–27,955)	9,812 (3,202–22,573)	7,590 (2,076–19,184)	21,679 (10,929-38,068)	6,958 (1,903-17,605)
	≥80	1,259 (153–4,488)	2,641 (547–7,563)	3,996 (1,097–9,981)	2,398 (291–8,448)	999 (25–5,466)	1,124 (28-6,136)	2,081 (253-7,355)
Total		4,033 (1,356–81,345)	15,029 (7,606–93,554)	18,402 (9,759–110,067)	30,775 (13,052–121,388)	38,638 (19,155–128,222)	50,621 (26,089-148,963)	73,994 (23,993-209,563)
Female								
Age, years	≤19	0 (0–32,403)	0 (0–32,403)	19,458 (2,382–65,370)	0 (0–34,112)	0 (0–35,036)	25,018 (3,072–82,321)	0 (0–49,902)
	20–39	0 (0–28,919)	0 (0–34,602)	0 (0–36,769)	0 (0–43,062)	0 (0–47,729)	0 (0–55,207)	21,206 (2,590–72,283)
	40–59	7,881 (957–27,996)	0 (0–13,672)	4,998 (127–27,333)	5,209 (132–28,469)	5,588 (141–30,496)	10,331 (1,255–36,510)	18,487 (5,074–46,234)
	60–79	8,536 (2,783–19,698)	3,461 (420–12,391)	7,887 (2,157–19,952)	11,562 (3,775–26,575)	8,306 (2,272–20,977)	14,605 (5,396–31,254)	7,302 (1,511–21,048)
	≥80	3,917 (811–11,240)	0 (0–5,555)	0 (0–6,908)	2,035 (52–11,088)	1,904 (48–10,391)	2,035 (52–11,088)	4,236 (515–14,901)
Total		20,333 (10,810–70,023)	3,461 (420–53,906)	32,343 (13,684–96,779)	18,807 (9,301–80,987)	15,799 (7,461–81,822)	51,989 (26,440–137,872)	51,232 (27,277–129,792)
All		24,367 (14,475–116,270)	18,490 (10,468–111,823)	50,746 (30,182–162,792)	49,562 (29,468–159,474)	54,437 (33,244–165,722)	102,610 (67,191–233,174)	125,226 (69,783–281,912)
**Tokyo**								
Male								
Age, years	≤19	0 (0–301,199)	0 (0–182,893)	0 (0–152,679)	0 (0–210,589)	14,776 (1803–457,538)	0 (0–383,404)	66,602 (1,684–307,796)
	20–39	31,328 (3,810–135,805)	0 (0–81,500)	48,084 (9,997–167,485)	32,056 (3,899–139,603)	39,383 (4,796–160,469)	61,263 (7,483–227,956)	104,464 (42,200–252,570)
	40–59	26,618 (8,680–67,969)	24,433 (9,698–62,639)	15,148 (3,131–58,768)	47,021 (21,853–106,125)	49,443 (24,049–104,190)	96,693 (53,586–174,249)	54,855 (26,230–116,634)
	60–79	12,901 (6,383–25,708)	21,119 (12,389–35,936)	36,065 (23,353–55,824)	20,214 (10,982–37,789)	16,911 (8,549–33,800)	40,031 (25,402–63,051)	25,561 (14,674–44,209)
	≥80	2,233 (775–7,691)	2,641 (547–8,574)	5,944 (2,578–13,749)	3,630 (1,205–11,839)	2,301 (702–9,670)	2,275 (707–9,489)	7,836 (3,551–16,965)
Total		73,080 (39,560–394,856)	48,193 (30,938–252,660)	105,242 (63,194–305,045)	102,921 (63,947–347,428)	122,816 (77,187–585,457)	200,263 (129,785–626,152)	259,318 (164,200–549,762)
Female								
Age, years	≤19	0 (0–497,300)	0 (0–176,264)	19,458 (2,382–240,627)	0 (0–238,281)	0 (0–323,981)	25,018 (3,072–524,559)	0 (0–205,949)
	20–39	28,141 (5,831–88,058)	24,843 (6,798–76,135)	43,042 (14,082–109,449)	47,724 (17,659–116,807)	44,741 (14,642–119,389)	70,352 (26,136–166,037)	67,469 (31,207–145,710)
	40–59	32,203 (14,408–69,825)	25,365 (9,348–57,667)	20,898 (7,377–58,355)	52,733 (26,601–100,166)	46,846 (21,690–96,059)	65,763 (33,183–123,739)	90,713 (53,680–149,251)
	60–79	21,403 (11,808–38,886)	22,126 (11,982–39,913)	30,618 (17,923–51,925)	35,416 (21,207–59,173)	28,254 (15,447–51,073)	39,634 (23,276–67,169)	40,726 (24,846–65,441)
	≥80	3,917 (811–13,897)	5,012 (1,036–15,990)	5,511 (1,140–17,991)	14,217 (6,284–30,794)	9,482 (3,673–23,818)	8,631 (3,037–23,979)	12,852 (5,572–29,742)
Total		85,663 (55,397–588,374)	77,345 (50,872–264,909)	119,527 (80,883–354,768)	150,090 (107,060–404,333)	129,323 (87,652–466,494)	209,297 (147,778–722,283)	211,460 (156,852–441,405)
All		158,743 (113,580–755,617)	125,539 (93,938–403,003)	224,769 (167,660–533,411)	253,011 (194,954–605,747)	252,138 (190,345–824,608)	409,659 (316,043–1,076,318)	470,778 (361,100–841,226)

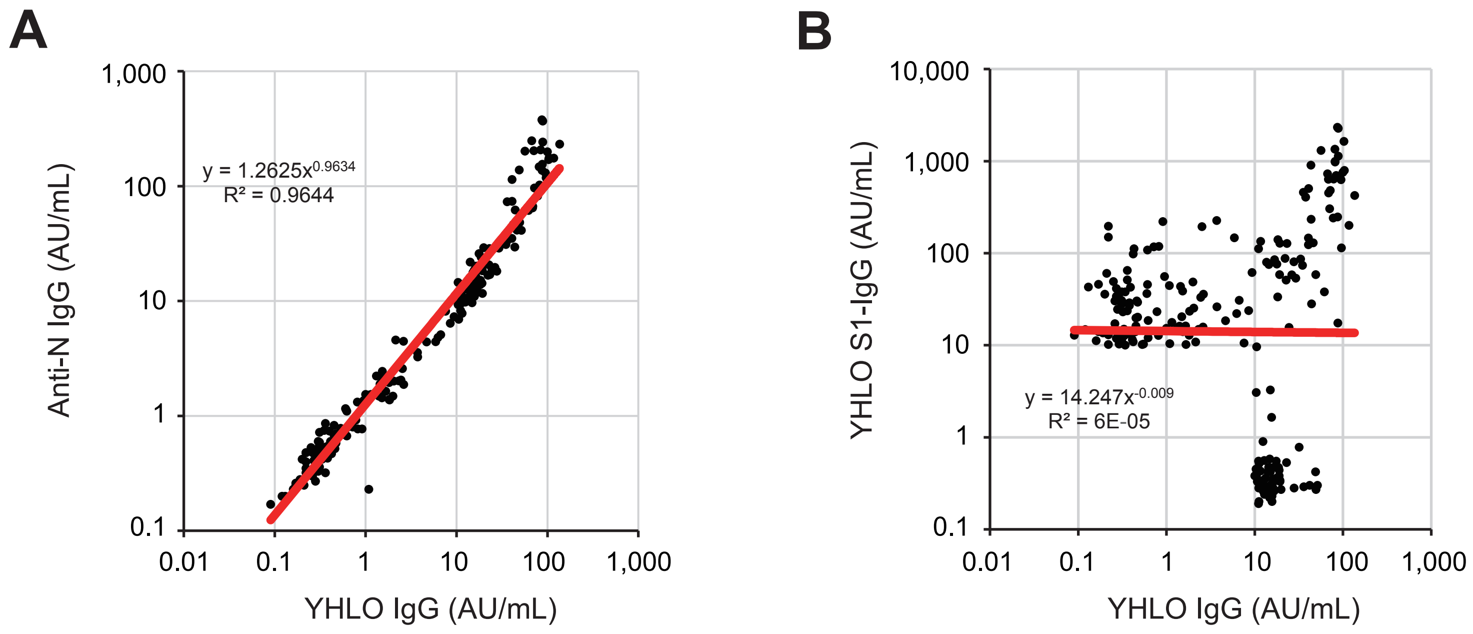

eFigure 1. Correlation of YHLO IgG level with anti-N IgG (a) or YHLO S1-IgG (b)

Correlation of YHLO IgG level with anti-N IgG (a) or YHLO S1-IgG (b). Each dot represents the level of YHLO IgG (x-axis), anti-N IgG (a, y-axis), or YHLO S1-IgG (b, y-axis) for an individual positive for YHLO IgG and/or YHLO S1-IgG. Red lines indicate regression curves. AU, Arbitrary units.

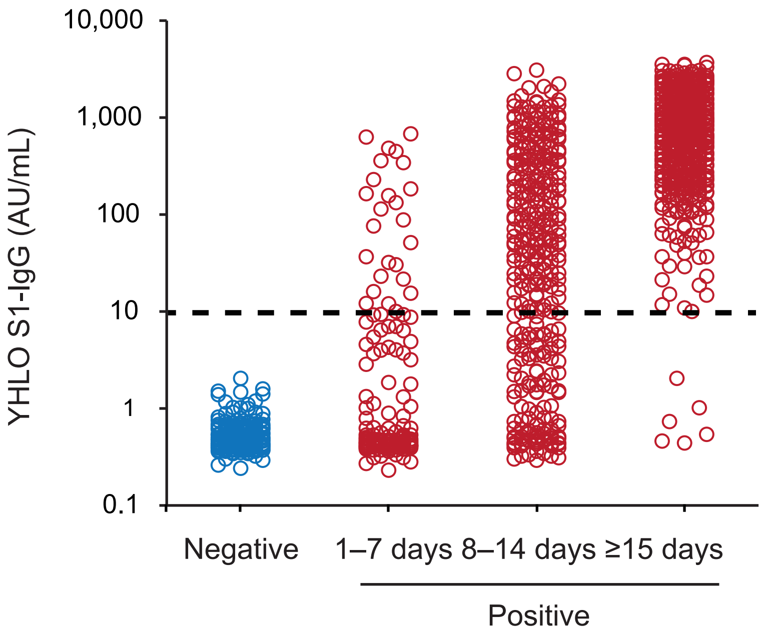

eFigure 2. YHLO S1-IgG levels in the PCR-negative and PCR-positve subjects
Each dot represents the level of YHLO S1-IgG in PCR-negative subjects (n=163), PCR-positive subjects 1 to 7 days after symptom onset (n=122), PCR-positive subjects 8 to 14 days after symptom onset (n=340), and PCR-positive subjects ≥15 days after symptom onset (n=423).
Broken line indicates the cutoff value (10 arbitrary units [AU]/mL).
